# Grand challenges and future oral epidemiology research

**DOI:** 10.3389/froh.2023.1349252

**Published:** 2024-01-10

**Authors:** Moréniké Oluwátóyìn Foláyan, Jacqueline R. Starr

**Affiliations:** ^1^ Department of Child Dental Health, Obafemi Awolowo University, Ile-Ife, Nigeria; ^2^ Oral Health Initiative, Nigerian Institute of Medical Research, Lagos, Nigeria; ^3^ Africa Oral Health Network, Alexandria University, Alexandria, Egypt; ^4^ Early Childhood Caries Advocacy Society, University of Manitoba, Winnipeg, MB, Canada; ^5^ Channing Division of Network Medicine, Department of Medicine, Brigham and Women’s Hospital, Boston, MA, United States; ^6^ Department of Medicine, Harvard Medical School, Boston, MA, United States; ^7^ Oral Health Policy and Epidemiology, Harvard School of Dental Medicine, Boston, MA, United States

**Keywords:** epidemiology, health policies, study designs, analytical methods, health disparities, one health, integration of oral health, community collaboration

## Introduction

Epidemiology is the study of the distribution and determinants of health outcomes. Descriptive epidemiology studies are conducted to evaluate the distribution of disease across different populations. The patterns that are identified as a result frequently inform etiologic hypotheses and contribute to health policies. Epidemiology tools—specialized study designs, statistical analytic methods, and an ability to understand and integrate strengths and limitations of the approach in interpreting the results—can be applied to study a wide range of factors, from climate change to social factors to molecular mechanisms.

Oral epidemiology is a subspecialty of epidemiology focusing on conditions and disease in the mouth, their distribution, and related factors and conditions. This article highlights opportunities and challenges in the field of oral epidemiology and defines the scope of this journal section. Among the many epidemiologic sub-disciplines, we concentrate here on five broad groupings: oral health disparities, social epidemiology, clinical epidemiology, molecular epidemiology, and epidemiologic methods.

### Oral health disparities

Amidst the many improvements in oral health for some populations or groups, oral health disparities persist (1) and are among the largest across various health indicators. These include unequal access to oral healthcare services and unequal oral health outcomes (2) attributable to socioeconomic backgrounds (3–7), geography—including urban vs. rural areas (8), race or ethnicity—with specific barriers to accessing culturally appropriate dental care (9), and age (7), to name a few dimensions of oral health inequality.

Oral health disparities and inequalities need to be addressed because they are unfair, unjust, and avoidable (10, 11). An equity-based and data-informed approach to health investment decision-making will provide a constructive framework for addressing service delivery disparities (12). First, more granular data are needed to identify inequalities across various dimensions, for example, race, ethnicity, gender or sexual identity, and geographic location, among others. Considering populations and communities to be uniform precludes the targeting of intervention strategies to meet the needs of specific sub-populations. Thus, data collection and analytic approaches that avoid aggregation are a necessary precursor to measure and address heterogeneity of health care access and oral health outcomes. Also critical is to enhance proficiency in data representation for diverse populations.

Second, oral health surveillance systems are needed to monitor oral health, inequities, and their temporal trends. These systems play a vital role in tracking indicators of equality, such as healthcare accessibility, educational achievement, income distribution, and representation in decision-making processes. Such data are also needed to implement effective oral health interventions (13). Unfortunately, the countries and populations that experience the greatest burden of disease and greatest inequity also have some of the weakest oral health surveillance systems globally (14).

Third, the incorporation of oral health research and oral disease control programs into well-established general health initiatives, can greatly enhance their effectiveness (15). Integration enables the pooling of resources, expertise, and data, leading to a more coordinated and holistic approach to overall health (16–18). There is lack of evidence regarding the cost-effectiveness of integrated oral and systemic health programs, and on sustainable models of integrated oral care in different contexts. There is also a lack of consistency regarding associations between oral and systemic health conditions, associations that could be more effectively identified through integrated oral and general health records and surveillance.

Fourth, established implementation frameworks and models, such as the Consolidated Framework for Implementation Research (19) or the RE-AIM framework (20), offer a systematic approach to understanding and addressing implementation challenges (21). A multidisciplinary approach will enable the dissemination of research findings to relevant stakeholders and facilitate the uptake of evidence-based practices through tailored messaging, stakeholder engagement, and effective knowledge exchange platforms.

### Social epidemiology

Social epidemiology concerns itself with multilevel and macro-level factors influencing the distribution of disease and its outcomes. It is a branch of epidemiology that extends its focus beyond individual-level factors to examine the intricate web of social and environmental determinants of oral health outcomes. Recognizing that health disparities and oral conditions cannot be fully understood or effectively addressed by examining only individual risk factors, social epidemiologists explore the multifaceted socio-ecological aspects that shape oral health and oral health disparities. Examples include exploration of how sociodemographic factors (e.g., socioeconomic status, education, employment, and income inequality) influence oral health; of how cultural norms, beliefs, and practices affect oral care, dietary habits, and tobacco use; of how geographic variation underlies access to fluoridated water, exposure to environmental toxins, and the availability of dental care facilities; and of how stress, social support, and other psychosocial factors may have bidirectional relationships with oral health conditions. Social epidemiologists place a strong emphasis on health equity and social justice. Researchers in this field advocate for policies and interventions that address the root causes of oral health disparities, striving for a more equitable distribution of resources and opportunities.

### Clinical epidemiology

As the subdiscipline concerned with clinical dentistry, clinical oral epidemiology encompasses studies of screening, diagnosis, effectiveness of treatment or prevention strategies, prognosis, and decision-making. Depending on practice setting and specialty, there is a breadth of high-priority research questions to inform clinical practice. In developed countries, dentists are seeking guidance on the use of new imaging modalities, including those incorporating machine learning and artificial intelligence (ML/AI). In resource-poor settings, technical innovation may be less critical than innovation in implementation. In all settings, there is an ongoing need for clinical trials to assess the safety, efficacy, and cost-effectiveness of materials, therapeutics, and procedures. In clinical epidemiology and across population-based research areas, many oral epidemiology subdisciplines require the development of standardized, agreed-upon, and validated measurements for different research purposes.

### Molecular epidemiology

Molecular epidemiology studies are designed to help identify molecular mechanisms of health and disease. Molecular epidemiology research often bridges wet lab and dry lab techniques, typically involving laboratory assays performed on human biospecimens. Such assays may target a single biomarker—for example, a single inflammatory cytokine or a specific bacterial pathogen. Or, as is increasingly the case, high-throughput techniques may be used to interrogate an entire complement of biomarkers, such the microbiome, the transcriptome, or the exposome. Not limited to dentistry or oral health, the molecular epidemiology literature is rife with unreproducible results. One reason for this is likely to be the multidisciplinary expertise required to conduct this work. For example, in oral microbiome research, microbiologists without methodologic training may lack expertise on study design and statistical analysis, and epidemiologists without microbiology training may lack the knowledge to ground their questions and interpretation of results. Under time pressure, multidisciplinary collaborations often involve transactional exchanges of data or information. To enhance rigor and reproducibility of research, and to go beyond studies of association to validation and experimentation, the field requires better-integrated, transdisciplinary collaborations.

### Epidemiologic methods

Research in any of the above areas is only as strong as the methods on which they are based, and each raises its own methodologic challenges. We especially welcome articles describing application or development of novel techniques to address such challenges, comparing different methodological approaches, highlighting concerns about commonly used methods, and discussing application of sound epidemiologic principles. We will also give high priority to research invoking modern causal inference frameworks, developing, and assessing the validity and reliability of innovative digital or ML/AI technologies, and applying multilevel modelling as appropriate, for example, for observations taken repeatedly from the same individuals (e.g., for different teeth or over time) or for data clustered geographically or socially.

Regardless of a manuscript's focus, we encourage all authors to explain why and how their results are meaningful despite potential limitations, for example, small sample size, sources of bias, potential confounding, measurement error or misclassification, among others. Inclusion of sensitivity analyses can enhance the clarity of explanations and often helps strengthen conclusions. Consistent with the journal's editorial principles, we also encourage the submission of papers irrespective of whether they yield statistically significant results. Results that can advance the field and are interpreted appropriately will be valued contributions.

We provide below a list of areas where we encourage manuscripts to help meet this grand challenge.

1. Investigations regarding oral health disparities or interventions to reduce disparities and their impact.
2. Implementation research. This could include an examination of barriers, facilitators, contextual factors, and strategies to promote evidence-based practices and to identify interventions that are effective in real-world practice.
3. Integration of oral health data collection into National Demographic Health Surveys in developing countries.
4. Reliability and validity of new imaging modalities, including those incorporating machine learning and/or artificial intelligence (ML/AI).
5. Molecular epidemiologic studies going beyond identification of associations to validation and experimentation regarding molecular mechanisms or biomarker applications.
6. Research on other potential sources of administrative data for oral diseases, with a particular emphasis on understanding the status of oral health policies and surveillance systems globally.
7. Generation of evidence that can help achieve the United Nations Sustainable Development Goals, which encompass a wide range of aims towards addressing social, economic, and environmental challenges.
8. Research embracing a “One health” approach, recognizing the interconnectedness of human, animal, and environmental health. Possible topics include zoonotic disease, environmental factors, and disease common to humans and animals.
9. Elucidation of associations between oral and systemic diseases, particularly through research designs and analyses aiming to eliminate confounding as a possible explanation of such relationships.
10. Work focusing on epidemiologic methods with special relevance to oral epidemiology or application to oral health factors or outcomes.
11. Explanations of how to apply state-of-the-art methods to oral epidemiology topics with a view towards improving the quality of oral epidemiology research and its communication. Examples include guidance on cost-effectiveness research, meta-analytic or synthetic reviews, or effective communication of research through tables and figures.
12. Establishment of standardized measurements of oral health or for specific oral diseases, or the incorporation of oral health into quality-adjusted life-year metrics.
13. Work involving collaborations with communities and advocates.
